# A pH-responsive Pickering Nanoemulsion for specified spatial delivery of Immune Checkpoint Inhibitor and Chemotherapy agent to Tumors

**DOI:** 10.7150/thno.46089

**Published:** 2020-08-07

**Authors:** Le Jia, Minghui Pang, Man Fan, Xuan Tan, Yiqian Wang, Menglin Huang, Yijing Liu, Qin Wang, Yanhong Zhu, Xiangliang Yang

**Affiliations:** 1 National Engineering Research Center for Nanomedicine, College of Life Science and Technology, Huazhong University of Science and Technology, Wuhan 430074, China.; 2 Hubei Key Laboratory of Bioinorganic Chemistry and Materia Medica, School of Chemistry and Chemical Engineering, Huazhong University of Science and Technology, Wuhan 430074, China.

**Keywords:** pH-responsive, Pickering nanoemulsion, immunogenic cell death, cancer chemo-immunotherapy

## Abstract

**Rationale:** Immune checkpoint (ICP) blockade therapy combined with chemotherapy is a promising treatment strategy for tumors. Chemotherapeutic agents usually function inside the tumor cells, while ICP inhibitors are efficacious out of the tumor cells. It is desirable to effectively co-deliver an ICP inhibitor and a chemotherapy agent to different sites of a tumor. We have designed an effective drug delivery system to accomplish both objectives.

**Methods:** We designed a Pickering nanoemulsion (PNE) using multi-sensitive nanogels with pH-responsive, hydrophilicity-hydrophobicity switch, and redox-responding properties as an oil/water interfacial stabilizer. The D/HY@PNE was employed for specified spatial delivery of the chemotherapy agent doxorubicin (DOX) and ICP inhibitor HY19991 (HY). We systematically investigated the pH-responsive disassembly of PNE, the release of DOX and HY from D/HY@PNE in the tumor microenvironment, enhanced tumor penetration of DOX, immunogenic cell death (ICD), antitumor efficacy, and the immune response induced by D/HY@PNE *in vitro* and *in vivo*.

**Results:** D/HY@PNE disassembled to release the ICP inhibitor HY and DOX-loaded nanogels due to the hydrophilicity-hydrophobicity reversal of nanogels in the acidic tumor microenvironment. Quantitative analysis indicates that D/HY@PNE presents enhanced tumor penetration behavior and effectively induces ICD. The strong immune response induced by D/HY@PNE was due to the efficient synergetic combination of chemotherapy and immunotherapy and resulted in enhanced antitumor efficacy in 4T1 tumor-bearing mice.

**Conclusion:** This novel strategy highlights the promising potential of a universal platform to co-deliver different therapeutic or diagnostic reagents with spatial regulation to improve the anti-tumor effect.

## Introduction

Immune checkpoint (ICP) blockade therapy efficacy is largely impaired by limited T-cell responses or immunologically “cold” tumors 1. Strategies, including induction of a robust T-cell response and reversing immune-suppressive microenvironment, enhance the response to immune checkpoint blockade therapy. Specific immunogenic chemotherapy not only kills tumor cells but also induces immunogenic cell death (ICD) to turn “cold” tumors “hot”, allowing tumor cells sensitization to immune checkpoint inhibitors 2, 3. Also, T cells recruited by ICD produce interferon-γ (IFN-γ) to help further eradicate tumor cells. However, prolonged IFN-γ signaling may also have an immunosuppressive role by promoting programmed cell death protein-ligand 1 (PD-L1) expression on tumor cells 4-6. PD-L1 interacts with programmed cell death protein 1 (PD-1), inactivating lymphocytes 7-9. Therefore, combination treatment of immunogenic chemotherapy agent DOX and PD-1/PD-L1 inhibitors can recruit T cells by blocking the PD-1/PD-L1 interaction, to achieve synergic therapy effect 7, 10.

However, DOX and PD-1/PD-L1 inhibitor need to be delivered to different sites of cells; DOX must be released inside the tumor cells while PD-1/PD-L1 inhibitor should be delivered out of the tumor cells to block the interaction between T cells and tumor cells. Previously, PD-1/PD-L1 antibodies and chemotherapy agents were generally administrated *via* different routes in mice with an intraperitoneal injection of PD-1/PD-L1 antibodies, while chemotherapy agents were intravenously injected 7, 11-13. Although PD-L1 is expressed mainly on tumor cells to regulate T-cell response, it is also expressed on other cells, such as macrophages. Therefore, non-targeted delivery of PD-1/PD-L1 inhibitors would decrease their accumulation in the tumor. Moreover, different administration routes used in the combination therapy were cumbersome to operate. Therefore, to develop delivering an ICP inhibitor and chemotherapy agent DOX to different sites of tumors in a single carrier is highly desirable.

In cold tumors, CD3^+^ and CD8^+^ T cell infiltrates are much lower at the tumor center than on the invasive margin 1. To facilitate T cell recruitment in the tumor center for the immunotherapy, sufficient ICD should be induced by chemotherapy 11, 14-18. Hence, chemotherapy agents with deep tumor penetration and enhanced tumor cell uptake are desired to achieve the maximum therapeutic efficacy 19. Many strategies have been developed to improve the tumor penetration and tumor cell uptake of nanomedicines in solid tumors, including surface modification, tumor specific size-switching, charge conversion, and dissociation of nanocarriers, etc. 19-24.

**Figure 1:**
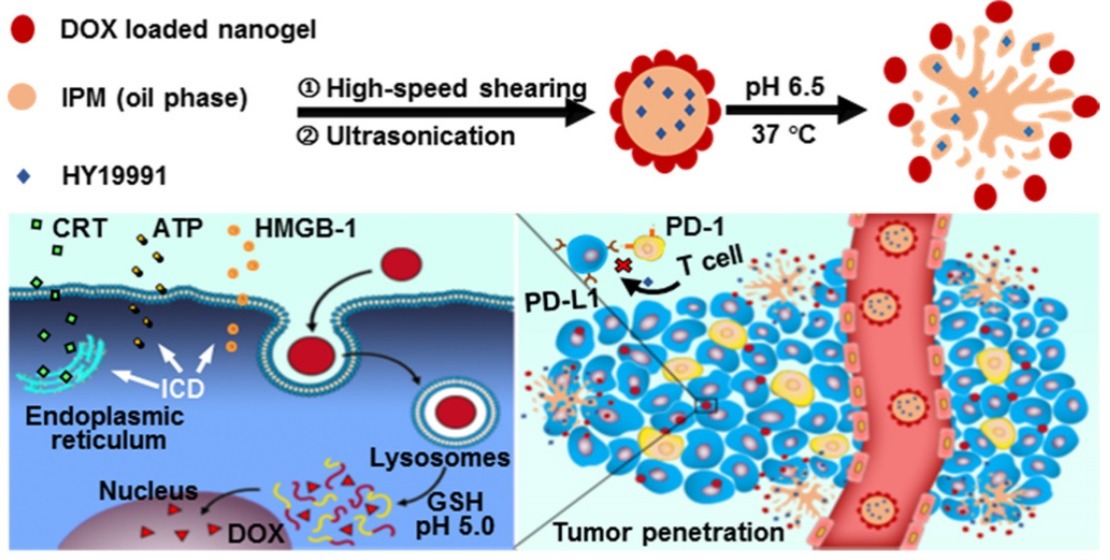
Schematic illustration of the preparation and disassembly of D/HY@PNE. In the tumor microenvironment, D/HY@PNE disassembled to release HY and DOX-loaded nanogels due to the hydrophilicity-hydrophobicity reversibility of the nanogels. The DOX-loaded nanogels had enhanced penetration ability and were easily internalized by tumor cells due to their small size and hydrophobicity. DOX was released from nanogels due to the high intracellular GSH concentration, triggering ICD. The released HY blocked the PD-1/PD-L1 connection between T cells and tumor cells, leading to the activation of T cells resulting in the synergistic therapeutic effect.

Based on our previous studies 25-27, herein, we constructed a pH-responsive Pickering nanoemulsion (PNE) by using multi-sensitive nanogels (designated as SNG) with pH-responsive, hydrophilicity-hydrophobicity switching, and glutathione (GSH)-responsive properties as oil/water interfacial stabilizers to co-deliver DOX and PD-1/PD-L1 inhibitor. Small-molecule PD-1/PD-L1 inhibitor HY19991 (structure shown in Figure S1A, ESI) was used in this study, which cuts off the interaction of PD-1/PD-L1 between T cells and tumor cells, resulting in the activation of T cells 28-30. HY19991 (denoted as HY) shows great clinical potential because of its lower cost and fewer side effects compared to clinically used PD-1/PD-L1 antibodies 31,32. Oil-soluble HY was encapsulated in the inner oil phase of the PNE and DOX was loaded in SNG as a shell layer. The resulting PNE was referred to as D/HY@PNE. In the normal physiological environment, the D/HY@PNE remained stable, but dissociated in the tumor matrix to release HY and DOX-loaded nanogels due to the hydrophilicity-hydrophobicity reversal of the nanogels (Figure 1). The penetration ability of DOX-loaded nanogels was enhanced, which were easily internalized by tumor cells due to their smaller size and hydrophobicity. DOX was released from GSH-responsive DOX-loaded nanogels due to the high intracellular GSH concentration, triggering ICD. The released HY then cut off the PD-1/PD-L1 connection between T cells and tumor cells, leading to the activation of T cells resulting in the synergistic therapeutic effect. This novel strategy may furnish a universal platform to co-deliver different therapeutic or diagnostic reagents with spatial regulation to improve the antitumor effect.

## Results and Discussion

### Synthesis and characterization of PNE

**Figure 2:**
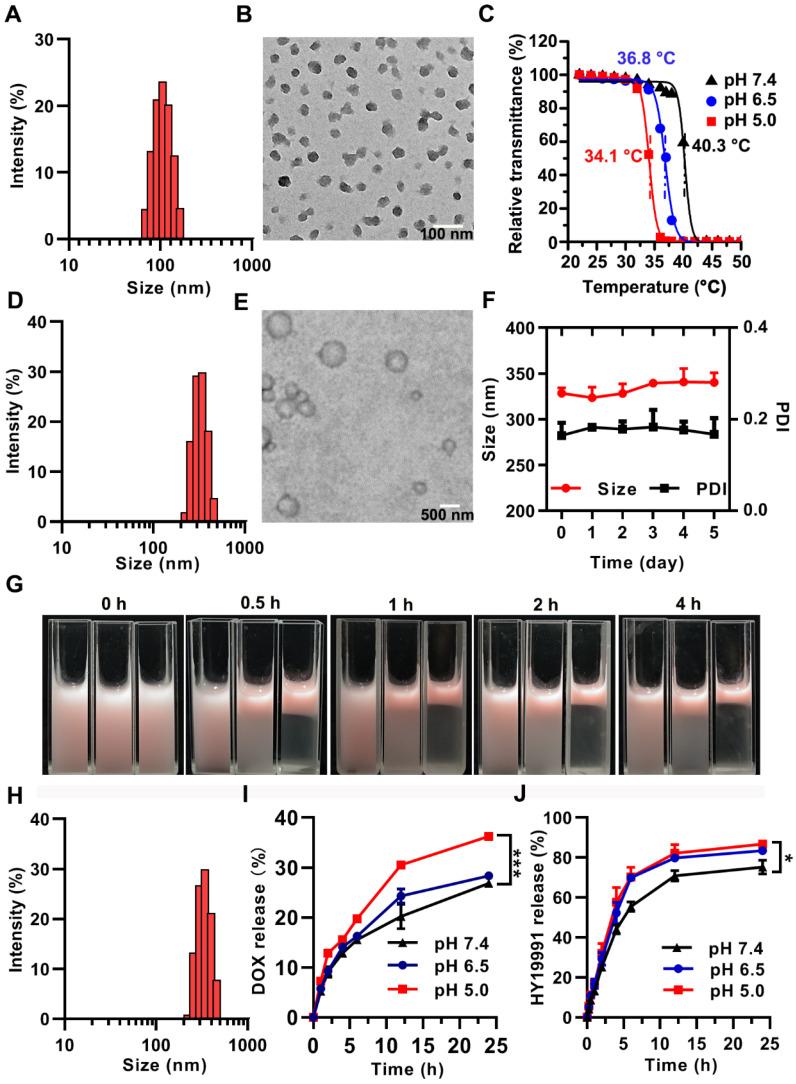
** Characterization of SNG, PNE and D/HY@PNE.** (**A**) Distribution of hydrodynamic diameter and (**B**) TEM image of SNG. (C) LCST measurement of SNG at different pH values by the transmittance analysis. (**D**) Hydrodynamic diameter and (**E**) TEM image of PNE. (**F**) Changes of the hydrodynamic diameter and PDI of PNE with time. (**G**) Photos of NR@PNE with different pH values and incubated at 37 °C for different times. From left to right, the pH value is 7.4, 6.5, and 5.0. (**H**) Hydrodynamic diameter of D/HY@PNE. (**I**) Cumulative released DOX and (**J**) HY19991 from D/HY@PNE at different pH values and time points; *p < 0.05, ***p < 0.001.

Pickering emulsions are stabilized by colloidal particles instead of conventional surfactants 33,34. When the droplet diameter is in the nano range, the emulsions are referred to as Pickering nanoemulsion 35,36. By sequential high-speed shearing and ultrasonic treatment, our laboratory successfully obtained PNE previously using nanogels 25. In this study, the multi-sensitive nanogels with pH-responsive, hydrophilicity-hydrophobicity switching, and GSH-sensitive properties were utilized as interfacial stabilizers to construct the intelligent PNE for spatial delivery of DOX and the ICP inhibitor (HY). The nanogels were prepared by the polymerization of thermosensitive *N*-isopropylacrylamide (NIPAM) as the main monomer, pH-responsive *N*-methylallylamine (MAA) and betaine-based zwitterionic sulfobetaine methacrylate (SBMA) as the comonomers, and disulfide bond-containing *N*, *N*′-bis(acryloyl) cystamine (BAC) as the cross-linker (Figure S1B, ESI). The lyophilized SNG could be readily re-suspended in water and showed a narrow hydrodynamic size distribution with a small particle diameter of 100 nm characterized by dynamic light scattering (DLS) (Figure 2A). The diameter of the dried SNG was about 40 nm by transmission electron microscope (TEM) (Figure 2B). SNG had pH-dependent lower critical solution temperatures (LCST) of 34.1 °C, 36.8 °C and 40.3 °C at pH 5.0, 6.5, and 7.4, respectively (Figure 2C). Thus, SNG was hydrophilic at pH 7.4 and switched to be hydrophobic at pH 6.5 or lower pH at physiological temperature (37 °C).

We selected isopropyl myristate (IPM), a common pharmaceutic adjuvant, as the oil phase due to its excellent biosafety. PNE (Figure S1C, ESI) was prepared by high-speed shear, followed by sonication in the presence of SNG as previously reported 25. The PNE (photo shown in Figure S2, ESI) acquired by this procedure exhibited a mean diameter of 327 nm (Figure 2D) with a polydispersity index (PDI) of 0.190 measured by DLS. TEM image (Figure 2E) showed that PNE was of spherical morphology, with a mean diameter of 360 nm. The mean size and PDI of PNE remained unchanged for over 5 days, confirming its good stability in the stock state (Figure 2F).

DOX was loaded into SNG by the solvent evaporation method to obtain DOX-loaded SNG used as an emulsion stabilizer instead of SNG. In addition, HY was added into IPM and the mixture was used as the oil phase. Using the procedure described above, D/HY@PNE was prepared and showed pink color due to the existence of DOX (Figure S3, ESI) with an average diameter of 337 nm and PDI of 0.160 (Figure 2H). The concentrations of DOX and HY in D/HY@PNE were 720 µg/mL and 667 µg/mL, respectively. D/HY@PNE was homogenous without phase separation at 4 °C for more than 6 months (Figure S4, ESI).

As mentioned above, a key feature of SNG was hydrophilic-hydrophobic conversion upon exposure to an acidic environment (≤pH 6.5). It is known that the balance of the hydrophilicity and hydrophobicity of surfactants is the most important factor for emulsion stability. When SNG turned hydrophobic, the PNE stabilized by SNG had the tendency to be unstable, resulting in phase separation. To observe the phase separation phenomenon, nile red (NR) was dissolved in IPM to prepare PNE. At 37 °C, PNE with NR in the oil phase was stable at pH 7.4, while gradually underwent the phase separation of water and oil (in red) at pH 6.5 and 5.0 (Figure 2G). Because of the hydrophobicity of SNG at low pH values, the phase separation rate at pH 5.0 was faster than that at pH 6.5. The pH-responsive stability of D/HY@PNE was also determined. At 37 °C, the phase separation of D/HY@PNE at pH 6.5 and 5.0 occurred within 0.5 h whereas D/HY@PNE at pH 7.4 was stable even for 4 h (Figure S5, ESI). D/HY@PNE released HY in the oil phase and DOX-loaded SNG gradually with the demulsification process. TEM images in Figure S6 showed that PNE at pH 6.5 gradually disintegrated at 37 °C, and the SNG removed from the oil-water interface into the water phase with similar morphology as the original SNG before emulsification.

The DOX and HY release profiles of D/HY@PNE were investigated in acidic conditions *in vitro* by the dynamic dialysis method. As displayed in Figure 2I, DOX release rate at pH 5.0 was faster than that at pH 6.5 and pH 7.4, likely attributed to the enhanced SNG hydrophobicity leading to the drug extrusion out of the networks of the nanogels. The cumulative release of DOX reached 36% at pH 5.0 and 28 % at pH 6.5 in 24 h. Also, the DOX release was about 46% at 10 mM GSH and pH 5.0 within 24 h (Figure S7, ESI), because the disulfide-bond in the nanogel crosslinker was cleaved under high GSH concentration. Figure 2J showed that the *in vitro* release of HY from D/HY@PNE at pH 6.5 and 5.0 was significantly faster than that at pH 7.4 due to the demulsification of D/HY@PNE with the cumulative release of HY reaching 70% at pH 5.0 while only 55% at pH 7.4 within 6 h. The accelerated HY release at acidic pH was probably due to the disassembly of D/HY@PNE.

### Cellular uptake assay

**Figure 3:**
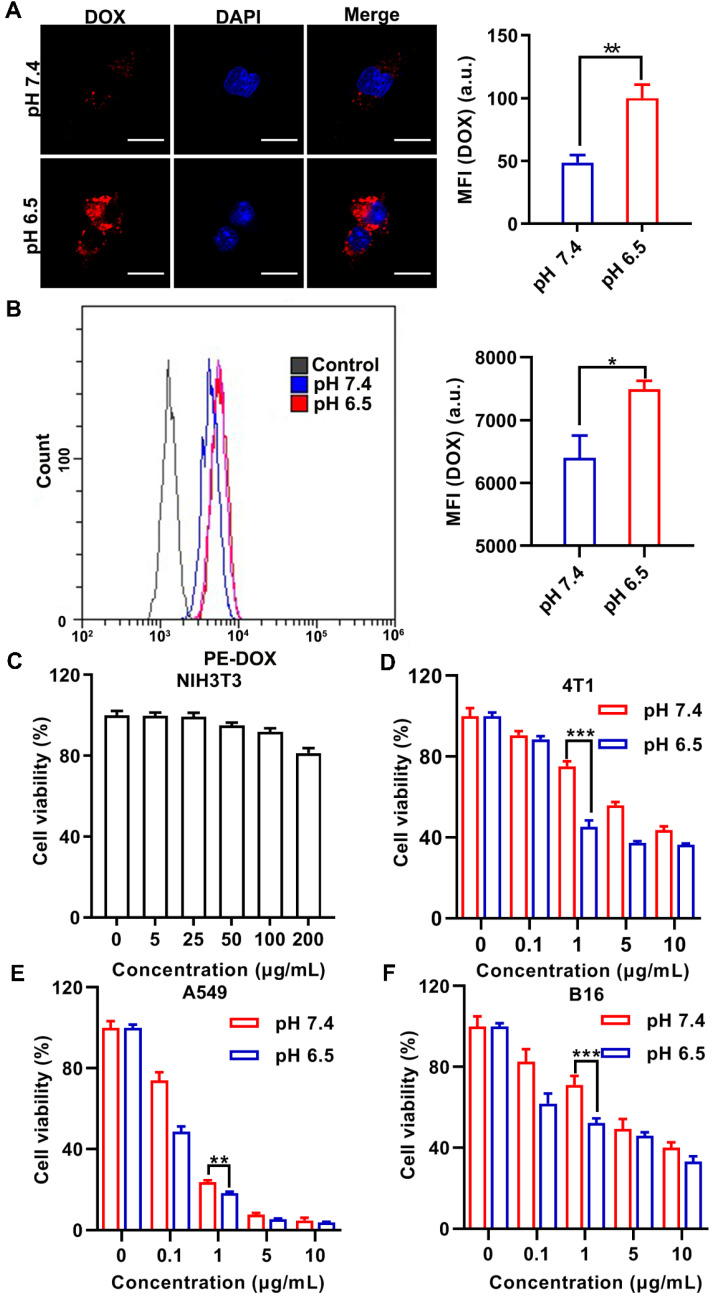
** Cellular uptake and viability analysis after various treatments.** (**A**) CLSM and (**B**) flow cytometry analyses of the internalization of D@PNE by 4T1 cells after 2 h of incubation at pH 7.4 or pH 6.5. The nucleus in Figure A was stained blue with 4',6-diamidino-2-phenylindole (DAPI). The scale bar in Figure A is 20 µm. (**C**) Viability of NIH-3T3 cells cultured with PNE for 24 h. The viability of (**D**) 4T1, (**E**) A549, and (**F**) B16 cells incubated with D@PNE was determined at pH 7.4 or pH 6.5 for 24 h; *p < 0.05, **p < 0.01, ***p < 0.001.

It has been reported that hydrophobic rather than hydrophilic nanomedicines are more readily internalized by cells 37-39. Given that HY interferes with the interaction between the T cells and tumor cells, D@PNE (PNE stabilized by DOX-loaded SNG) was incubated with murine breast-cancer cells (4T1) for monitoring cell uptake at pH 7.4 and 6.5 for 2 h. Cellular distribution of DOX was evaluated by confocal laser scanning microscopy (CLSM) and flow cytometry. As shown in Figures 3A and 3B, D@PNE was internalized in 4T1 cells at pH 6.5 with a relatively stronger DOX fluorescence than that at pH 7.4. This difference may be attributed to the alteration of SNG to hydrophobicity at 37 °C in the pH 6.5 culture medium, facilitating the interaction of SNG with the cell membrane and enhancing internalization 38, 39. In contrast, D@PNE remained stable and was hydrophilic at 37 °C in the pH 7.4 culture medium, which hampered the cellular internalization to some extent. The data suggested that D@PNE could be better internalized by tumor cells in the acidic tumor microenvironment.

### Cytotoxicity assay

The PNE at different concentrations (based on nanogels concentration) was incubated with the mouse fibroblast (NIH-3T3) and 4T1 cells for 24 h and the relative cell viability was evaluated by the cell counting Kit-8 (CCK-8) assay. As expected, PNE showed good biocompatibility and the viability of both cell lines was above 80% (Figure 3C and S8 in ESI). Subsequently, D@PNE cytotoxicity in 4T1, human non-small cell lung cancer (A549), and murine melanoma (B16) cell lines at pH 7.4 and 6.5 was investigated. D@PNE inhibited the proliferation of 4T1, A549 and B16 cells in a concentration dependent manner (Figure 3D-F). Half-maximal inhibitory concentrations (IC_50_) of D@PNE for 4T1 cells at pH 7.4 and pH 6.5 were 7.2 µg/mL and 2.0 µg/mL respectively. The IC_50_ of D@PNE for A549 cells was 0.3 µg/mL at pH 7.4 and 0.2 µg/mL at pH 6.5. The IC_50_ of D@PNE for B16 cells was 1.1 µg/mL at pH 6.5, lower than that at pH 7.4 (IC_50_=4.8 µg/mL). After a 24 h treatment with different concentrations of D@PNE, IC_50_ values at pH 6.5 were lower than at pH 7.4 in all cells. These data further demonstrated more efficient internalization of D@PNE by tumor cells and stronger cytotoxicity at pH 6.5 than at pH 7.4 due to the hydrophobicity of SNG at acidic pH. At the same concentration, the killing effect of D@PNE in A549 was superior to the other two cell lines, which may be attributed to the different sensitivity of cells to DOX.

### ICD induced by D@PNE *in vitro*

**Figure 4:**
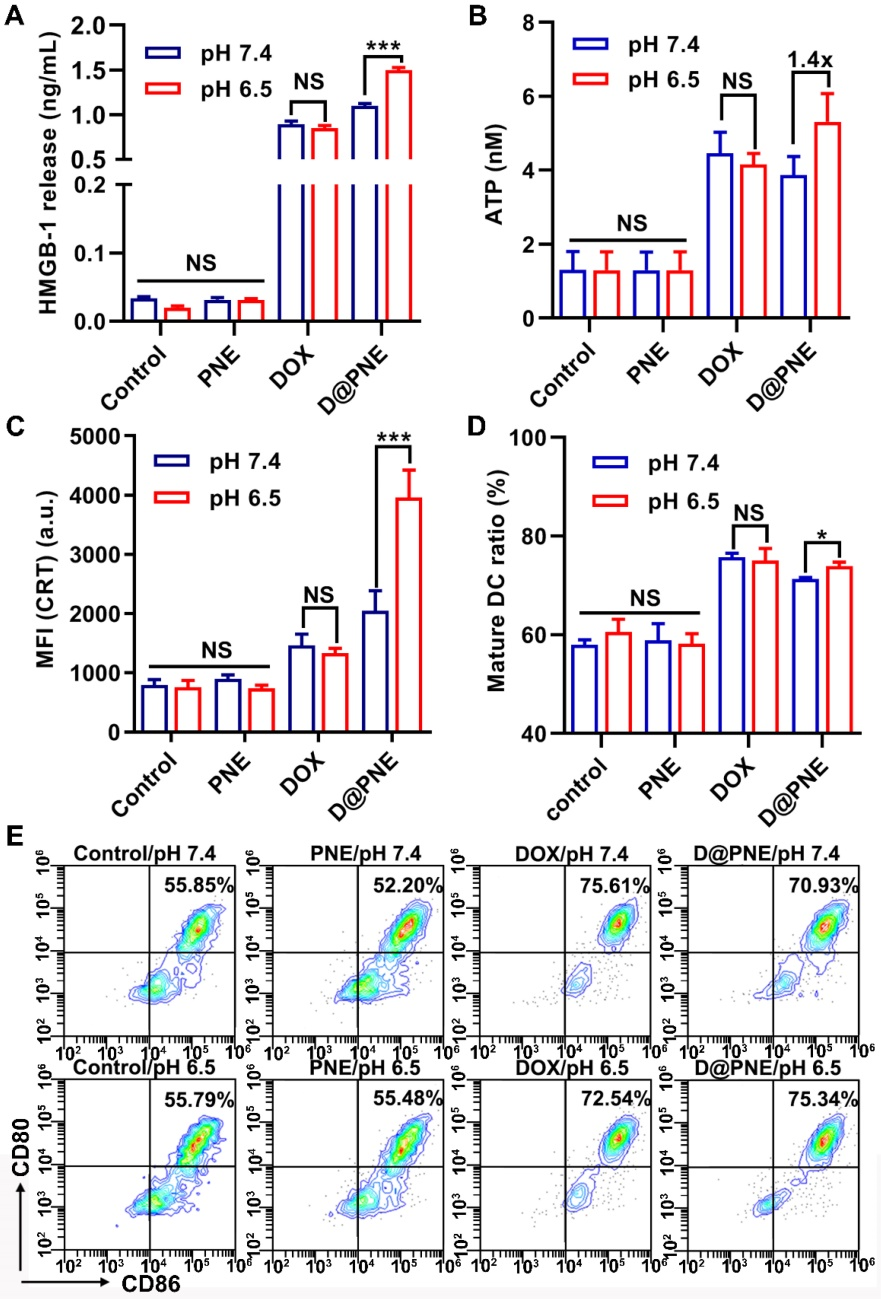
** D@PNE effectively induced ICD of 4T1 cells and elicited DC maturation* in vitro.*** (**A**) Extracellular HMGB-1 release and (**B**) ATP secretion of 4T1 cells with different treatments for 2 h. (**C**) CRT exposure of 4T1 cells with different treatment for 4 h. (**D**) Quantification and (**E**) representative flow cytometry plots of the mature DCs (CD11c^+^CD80^+^CD86^+^) after various treatments *in vitro*. The DOX concentration was 1µg/mL; *p < 0.05, **p < 0.01, ***p < 0.001.

As an immunogenic chemotherapy agent, DOX was reported to induce ICD in many cancer cell lines 10, 17, 40. As illustrated in Figure S9, the ICD of tumor cells is characterized by cell surface expression of calreticulin (CRT), extracellular secretion of high mobility group box 1 (HMGB-1), and extracellular release of adenosine triphosphate (ATP) 10, 16, 17, 41. Figures 4A and 4B show that treatment with PNE at both pH values, HMGB-1 release and ATP secretion were not significantly different from the control group, whereas markedly increased after treatment with DOX and D@PNE. HMGB-1 release and ATP secretion induced by DOX at pH 7.4 were slightly higher than those at pH 6.5, but there was no significant difference between the two groups. The supernatant HMGB-1 content was also significantly enhanced after D@PNE treatment at pH 6.5 compared to pH 7.4. Treatment of D@PNE at pH 6.5 induced 1.4-fold higher extracellular secretion of ATP than at pH 7.4. CRT secretion was efficiently enhanced after stimulation with DOX and D@PNE for 4 h, whereas no significant difference could be detected in PNE-treated cells compared to the control cells. CRT secretion was 1.94-fold higher at pH 6.5 than at pH 7.4 after stimulation with D@PNE, while no significant difference was observed between pH 6.5 and 7.4 after DOX treatment (Figure 4C). The data demonstrated that the pH-responsive D@PNE induced better ICD effects. The higher ICD-induction efficacy of D@PNE may be attributed to the increased cellular uptake of DOX-loaded SNG from the demulsification of D@PNE.

To further evaluate D@PNE-induced immunogenicity, we investigated ICD-induced DC maturation (CD11c^+^CD80^+^CD86^+^). Bone marrow derived dendritic cells (BMDCs) isolated from BALB/c mice were incubated with D@PNE-pretreated tumor cells, and then examined using flow cytometry. Compared to the control, PNE treatment did not induce DC maturation, which was comparable between the control and PNE-treated cells (Figures 4D and 4E). However, DC maturation was efficiently induced after incubation with DOX and D@PNE-pretreated tumor cells for 24 h. There was no significant difference of DC maturation between pH 6.5 and pH 7.4 in the DOX groups. However, D@PNE-pretreated tumor cells at pH 6.5 significantly promoted the maturation of DCs, which was 1.22-fold compared to the control group, possibly due to the increased ICD of tumor cells. This suggested that the higher ICD-induction efficacy of D@PNE at pH 6.5 elicited more DC maturation.

### Enhanced tumor penetration of D/HY@PNE

**Figure 5:**
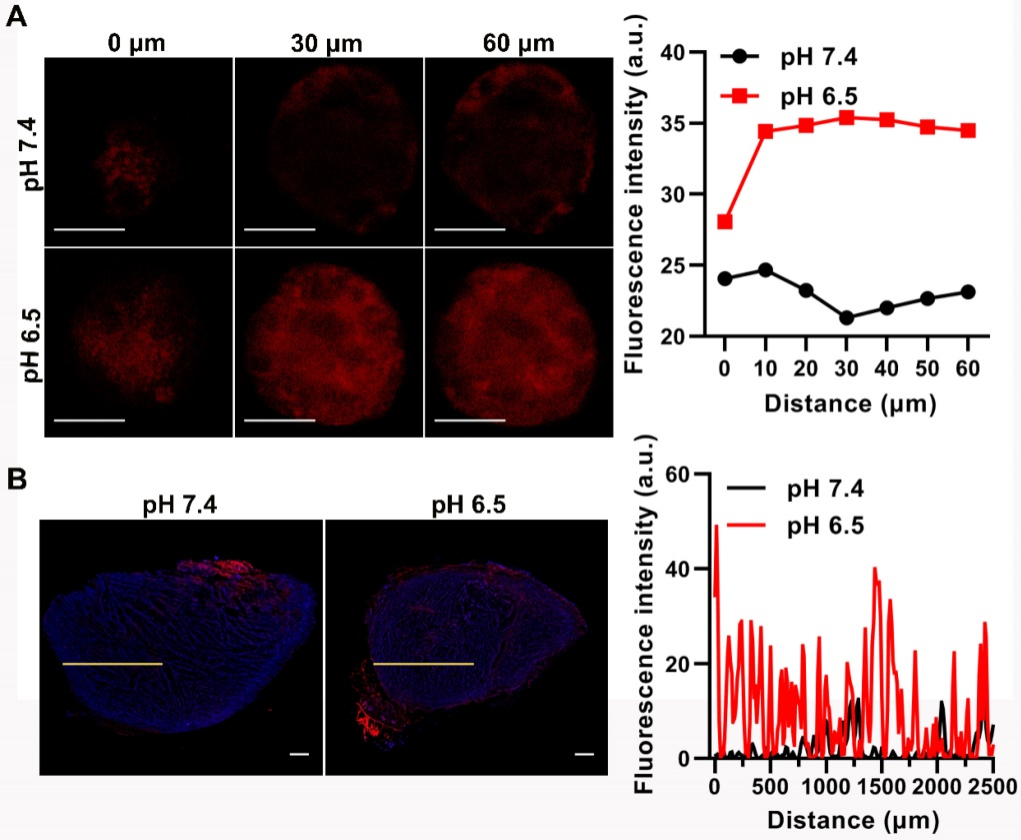
** Tumor penetration behavior of D/HY@PNE.** (**A**) Representative CLSM images of 4T1 MCSs after 5 h incubation with D/HY@PNE at 5 µg/mL DOX at pH 7.4 or 6.5 at 37 °C. The scale bar is 20 µm. DOX fluorescence intensity at different depths is shown in the right panel. (**B**) Fluorescent microscopic images of 4T1 tumor tissue sections at 2000 µm incubated with D/HY@PNE at DOX concentration of 5 µg/mL at pH 7.4 or 6.5 for 24 h at 37 °C. The scale bar is 500 µm. The distribution profile of DOX fluorescence intensity from the edge of the tumor sections on the specified yellow line is shown in the right panel.

Small-size nanomedicines usually penetrate deeper into the tumor than those with bigger size 42, 43. In our study, PNE disintegrated at pH 6.5 and 37 °C. SNG with the original shape and size almost entirely removed from the oil-water interface and entered into the aqueous phase. As shown in Figures 2A and 2D, the size of SNG was smaller than PNE. Therefore, the sensitivity of PNE in response to acidic tumor microenvironment to release smaller nanogels prompted us to investigate its tumor penetration ability. We used 4T1 multicellular spheroids (MCSs) as an* in vitro* model to assess the penetration of D/HY@PNE. MCSs were incubated with D/HY@PNE for 5 h at pH 7.4 and pH 6.5 and subsequently subjected to CLSM Z-stack scanning. At the scanning depth of 30 μm, the DOX fluorescence intensity in the interior area of the MCSs at pH 7.4 dropped considerably in contrast to that at pH 6.5. As is evident from Figure 5A, the red fluorescence inside the MCSs was observed even at the scanning depth of 60 µm, demonstrating the deep penetration of D/HY@PNE because of their rapid disintegration into smaller DOX-loaded SNG at pH 6.5. In contrast, D/HY@PNE showed limited penetration due to their large size and stability at pH 7.4. The tumor penetration capability was further investigated by incubating *ex vivo* tumor tissues in cell culture media containing D/HY@PNE for 24 h at different pH values. Consistent with the MCS results, a greater amount of D/HY@PNE penetrated into the *ex vivo* tumor tissues at pH 6.5 than at pH 7.4 (Figure 5B).

### Antitumor efficacy of D/HY@PNE in 4T1 tumor-bearing mice

**Figure 6:**
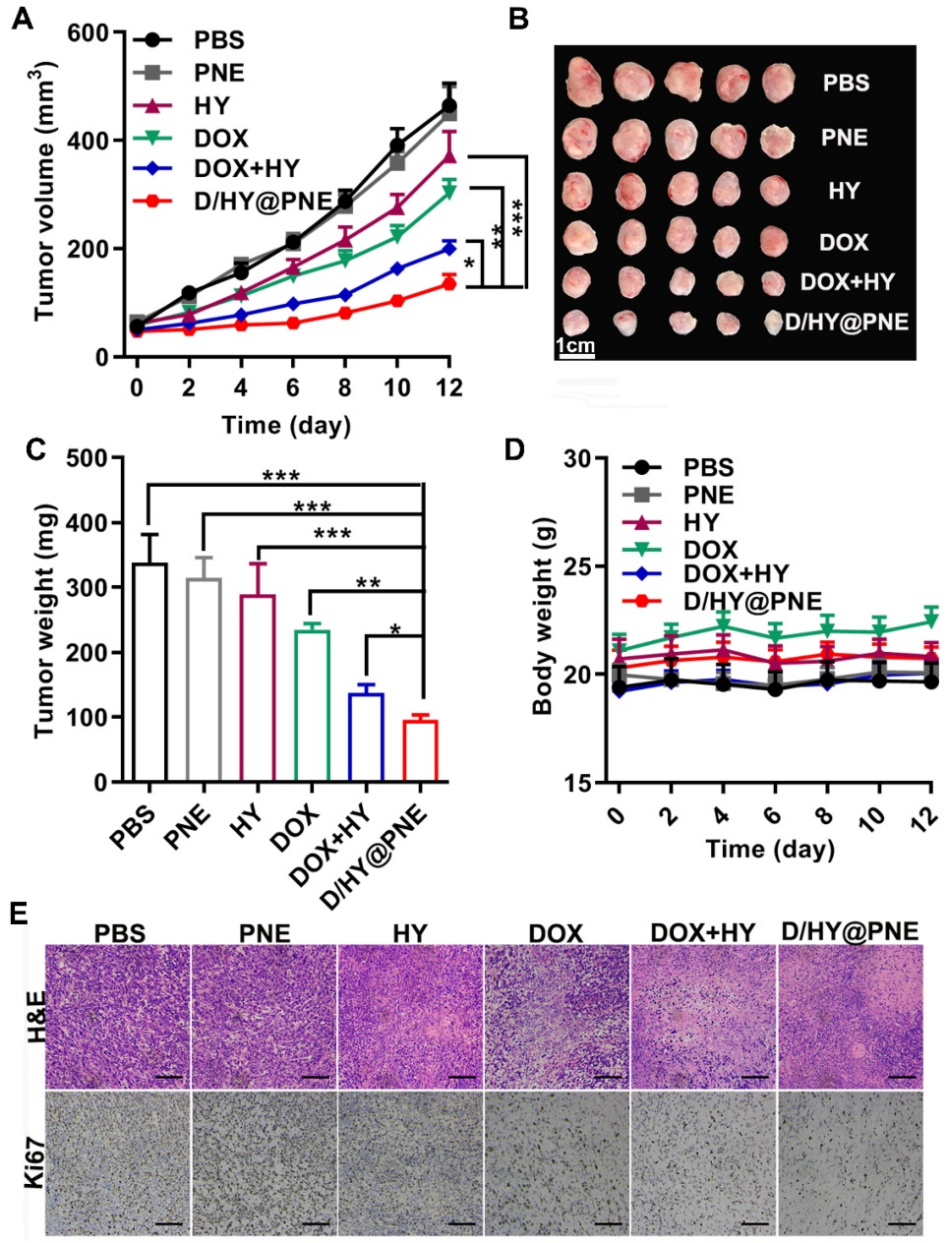
** Antitumor efficacy of D/HY@PNE *in vivo*.** (**A**) Tumor growth curves in 4T1 tumor-bearing mice after various treatments. (**B**) Images of the tumors at the end of antitumor studies. (**C**) Tumor weight at the end of various treatments. (**D**) Body weight of 4T1-bearing mice. (**E**) H&E and Ki67 analysis of tumor tissues with various treatments. The scale bar is 50 µm; *p < 0.05, **p < 0.01, ***p < 0.001.

The *in vivo* antitumor efficacy of D/HY@PNE was examined in 4T1 tumor-bearing mice. All treatments including phosphate-buffered saline (PBS), PNE, free DOX, free HY, DOX+HY, and D/HY@PNE were injected *via* intratumoral administration on day 0, 2, and 4. Based on the volume, images, and weight of the tumors, either DOX or HY alone showed moderate antitumor effects (Figure 6A-C), and the combination therapy with free DOX and HY showed a greater therapeutic efficacy than the monotherapy. However, about 71% inhibition after treatment with D/HY@PNE, exhibited the best antitumor effect. Furthermore, hematoxylin and eosin (H&E) staining and immunohistochemistry staining of Ki67 (cell proliferation marker) showed the largest damaged area in tumor tissues and a significant decrease in cell proliferation after treatment with D/HY@PNE (Figure 6E). The body weights of mice had no obvious changes during the 12 days of treatment (Figure 6D). Also, H&E staining showed no significant physiological changes in major organs including the heart, liver, spleen, lung, and kidney (Figure S10, ESI), suggesting good biocompatibility of D/HY@PNE.

### Immune response induced by D/HY@PNE *in vivo*

**Figure 7:**
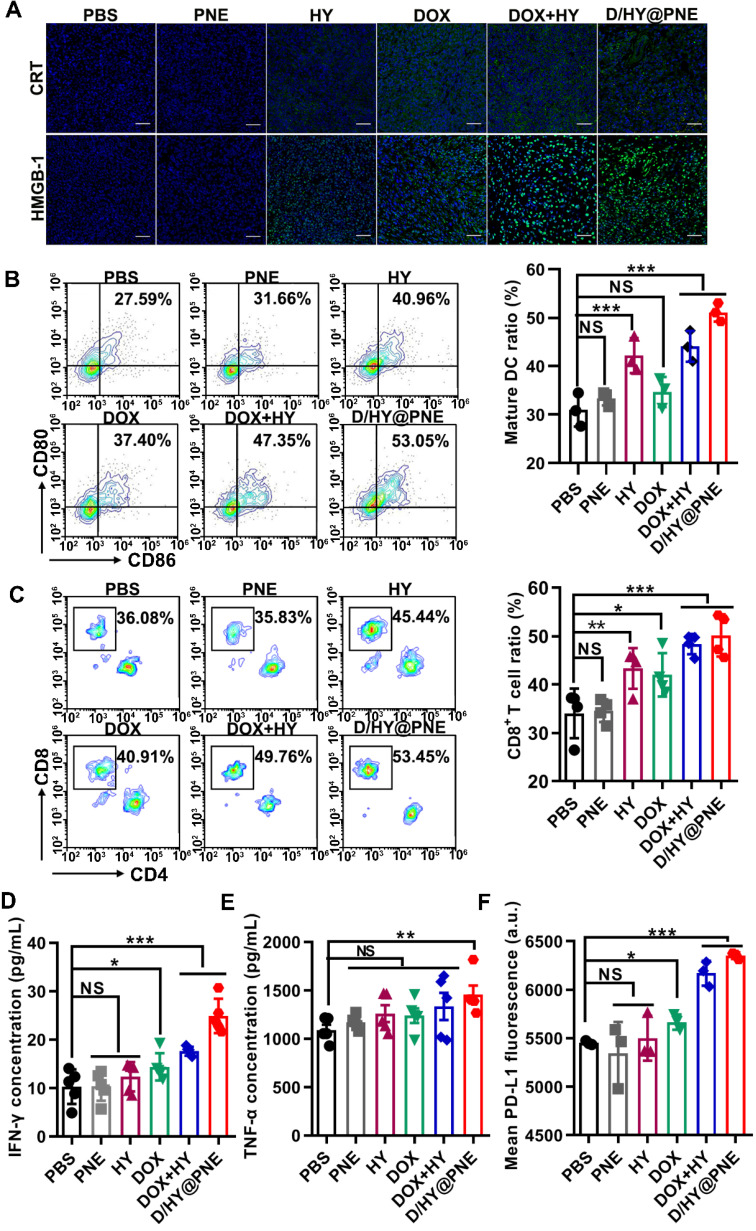
** Immune response induced by D/HY@PNE *in vivo*.** (**A**) Immunofluorescence assay of CRT and HMGB-1 in the tumor tissues after various treatments. The scale bar is 50 µm. (**B**) Representative flow cytometry plots showing mature DCs (CD11c^+^CD80^+^CD86^+^) after various treatments. Absolute quantification of mature DCs is shown in the right panel. (**C**) Representative flow cytometry plots showing cytotoxic T lymphocytes (CD3^+^CD8^+^) after various treatments. Absolute quantification of cytotoxic T lymphocytes is shown in the right panel. (**D**) ELISA analysis of INF-γ and (**E**) TNF-α levels in sera. (**F**) Flow cytometry analysis of the PD-L1 expression level in the tumor tissues after various treatments; *p < 0.05, **p < 0.01, ***p < 0.001.

We investigated the immune response underlying the outstanding antitumor effects of D/HY@PNE by examining whether the nanoemulsion induced ICD of tumor cells *in vivo*. Tumors were harvested on day 12 after the first treatment and CRT and HMGB-1 immunofluorescence assays demonstrated more abundant expression after combination treatments with free DOX and HY as well as D/HY@PNE (Figure 7A).

ICD induced by D/HY@PNE could activate the immature DC. To clarify the immune response of D/HY@PNE induced ICD, the tumor-draining lymph nodes were harvested on day 3 after the first treatment for the detection of DC maturation using flow cytometry. As shown in Figure 7B, both HY or DOX could induce DC maturation, but the combination treatment of DOX and HY showed better DC maturation induction. As expected, the highest DC maturation of 53.1% was observed in the group treated with D/HY@PNE which was 1.48-fold higher than the DOX group and 1.65 times higher than the PBS group.

It has been reported that mature DCs can activate antitumor immunity through endocytosis presenting tumor antigens to T cells 44. We analyzed the intratumoral infiltration of T lymphocytes by flow cytometry (Figure 7C). There was no significant difference between the PNE and PBS groups. The proportions of tumor infiltrating CD8^+^ T cells in the DOX and HY groups were 1.23- and 1.27-fold higher than the PBS group. Compared to DOX or HY alone, the ratio of CD8^+^ T cells was greatly increased after combination treatment with free DOX and HY. D/HY@PNE treatment exhibited 53.5% CD8^+^ T cell intratumoral infiltration, which was 1.47-fold higher than the PBS group, suggesting that D/HY@PNE group recruited more infiltrating CD8^+^ T cells and induced a more robust immune response. The levels of CD8^+^ T cells, myeloid-derived suppressor cells (MDSCs), and regulatory T cells (Tregs) in tumor tissues were also monitored by immunofluorescent staining. Consistent with flow cytometry results, the number of CD8^+^ T cells increased in the D/HY@PNE group compared to the other five groups (Figure S11, ESI). However, immunosuppressive cells, such as MDSCs and Tregs, were decreased in the D/HY@PNE group compared to the other five groups.

Active CD8^+^ T cells secrete cytokines, including IFN-γ and tumor necrosis factor α (TNF-α), to inhibit tumor cell growth 45, 46. Therefore, we analyzed serum levels of IFN-γ and TNF-α and found no significant difference in both cytokine levels between PNE and PBS groups (Figures 7D and 7E). Both DOX and HY groups showed slightly higher serum levels of IFN-γ and TNF-α than the PBS group. The concentrations of IFN-γ and TNF-α in combination treatments of DOX and HY were higher than that of DOX or HY alone. D/HY@PNE induced the secretion of IFN-γ and TNF-α efficiently, which were 2.41-fold and 1.33-fold higher, respectively, than in the PBS group. The results suggested that D/HY@PNE induced a better antitumor immune response.

IFN-γ acts as a “double-edged sword” in the antitumor immune response. Long-term expression of IFN-γ in tumor tissues upregulates the expression of PD-L1 on the tumor cells, resulting in immunosuppression to promote tumor growth 4. PD-L1 inactivates lymphocytes after interacting with PD-l present on the surface of various immune cells 7, 9. We evaluated the expression of PD-L1 in tumor tissues by flow cytometry. Figure 7F showed that the PD-L1 expression in tumor tissues increased after DOX-involved formula treatment compared with PBS, suggesting that DOX initiated an immune response as well as elevated the expression PD-L1 on tumor cells. Thus, high expression PD-L1 on tumor cells induced immune suppression to abate the therapy effects. To block PD-L1 on tumor cells induced by DOX, HY synergetic combination with DOX impaired the immune checkpoint blockade to activate T cells.

## Conclusion

We developed a pH-responsive Pickering nanoemulsion (D/HY@PNE) by using intelligent nanogels as oil/water interfacial stabilizers for a synergistic combination of chemotherapy and immunotherapy. In the acidic tumor microenvironment, D/HY@PNE disassembled to release ICP inhibitor HY and DOX-loaded nanogels due to the hydrophilicity-hydrophobicity reversal of the nanogels, permitting deep tumor penetration and effectively inducing ICD. The strong antitumor immune response induced by D/HY@PNE enhanced antitumor efficacy in 4T1 tumor-bearing mice. In this study, we considered using a co-loaded carrier to deliver chemotherapy agent DOX and immunotherapy agent HY to different sites with feasible operation, whereas the more delicate carrier can be fabricated for spatial and temporal delivery of different therapeutic reagents.

## Experimental Section

### Materials, cells, and animals

NIPAM (purity>98.0%) was purchased from Tokyo Chemical Industry Co., Ltd. (Tokyo, Japan) and recrystallized from n-hexane. BAC, SDS, IPM, and DAPI were obtained from Sigma-Aldrich (St, Louis, USA). PBS, Dulbecco's Modified Eagle's Medium (DMEM), RPMI Medium 1640 and Penicillin-Streptomycin solution were obtained from GE Healthcare Life Sciences HyClone Laboratories (Logan, UT, USA). Fetal bovine serum (FBS), Trypsin-EDTA and collagenase type I were bought from Gibco (USA). Doxorubicin hydrochloride (DOX·HCl) was purchased from DaLian Meilun Biotechnology CO., Ltd. (DaLian, China). HMGB-1 ELISA kit, IFN-γ ELISA kit, and TNF-α ELISA kit were acquired from MSKBIO (Wuhan, China). CCK-8 assay kit was purchased from Dojindo (Japan). ATP bioluminescence assay kit was obtained from Beyotime Institute of Biotechnology (Shanghai, China). Antibodies were purchased from Bio Legend, Inc. (San Diego, USA).

NIH-3T3, 4T1, A549, and B16 cells were from the cell bank of the Chinese Academy of Sciences (Shanghai, China) and maintained in our laboratory. NIH-3T3 cells were cultured in DMEM containing 10% FBS and 100 U/mL penicillin and 100 µg/mL streptomycin. 4T1 cells and A549 cells were cultured in RPMI-1640 medium with 10% FBS and 100 U/mL penicillin and 100 µg/mL streptomycin. Cells were incubated at 37 °C in a humidified atmosphere with 5% CO_2_.

Female Balb/c mice were purchased from Changsheng Biotechnology Co., Ltd (Liaoning, China) and housed in a specific pathogen-free environment. All animal experiments were carried out according to the protocols approved by the Institutional Animal Care and Use Committee of Huazhong University of Science and Technology (Wuhan, China).

### Preparation of PNE and D/HY@PNE

SNG was prepared as described in our recent study 26. The aqueous phase of nanogels (2 wt %) was obtained by placing lyophilized SNG powder into ultrapure water at 4 °C overnight. IPM (0.3 mL) as oil phase was gradually added into SNG dispersion (2 wt%, 2.7 mL), and then was subjected to shearing at 13000 rpm for 5 min using a homogenizer (FA25, Fluko, Germany) to obtain a preliminary emulsion. The stable PNE was obtained by sonicating the above emulsion (250 W, 10 s:10 s, 5 min) in an ice-water bath using an ultrasonic homogenizer (JY92-IIN, SCIENTZ, China). DOX-loaded SNG was obtained by the organic solvent evaporation method 26. D@PNE was prepared using DOX-loaded SNG as a Pickering nanoemulsion stabilizer. In addition, 2 mg of HY dissolved in 0.3 mL of IPM was adopted as an oil phase instead of pure IPM. D/HY@PNE was obtained by a similar method used for the preparation of PNE and D@PNE.

### Characterization

The morphology of SNG and PNE was characterized by TEM. SNG and PNE were diluted ten times with ultrapure water and dripped onto a carbon-coated copper grid, respectively. The samples were dyed with 1 wt% phosphotungstic acid solution for 30 seconds and washed with ultrapure water to remove superfluous phosphotungstic acid. After the sample was dried in the air, photographs were taken by TEM (Tecnai G2-20, FEI Corp., Netherlands) at 120 kV. The hydrodynamic diameters and size distribution of the samples were measured by DLS (Zetasizer Nano ZS90, Malvern Instruments, U.K.) at 25 °C. The average particle size was expressed based on intensity.

The LCST of SNG was measured by transmittance analysis. The SNG powder was dispersed in PBS at a concentration of 10 mg/mL at different pH values (7.4, 6.5 or 5.0). The transmittance of the SNG dispersions was measured at 560 nm at the designated temperature from 22 to 50 °C after balancing for 5 min at every measuring point by UV-vis spectrophotometer (UV 3600, Shimadzu, Japan). Boltzmann fitting was applied and the valley of the differentiation curve was defined as the LCST.

To detect pH-responsive stability of PNE, nile red was dissolved in IPM to prepare NR@PNE. The NR@PNE was diluted ten times with PBS at pH 7.4, 6.5 and 5.0, and placed in a water-bath at 37 °C. Photographs were taken at different times to show the phase separation at different pH values. The pH-responsive stability of D/HY@PNE was detected following the same procedure. PNE was also diluted 10 times with PBS at pH 6.5, and placed in a water-bath at 37 °C for 4 h for de-emulsification. The morphology of nanogels in the aqueous phase after disassembly of PNE was detected by TEM.

The drug loading capacity (DLC) of the DOX-loaded SNG was determined to be 4.0%, which was consistent with a previous report 26. The concentrations of DOX and HY in D/HY@PNE were determined according to the amount of DOX-loaded SNG and HY.

### Drug release studies

The release behavior of DOX and HY from D/HY@PNE *in vitro* was determined by the dynamic dialysis method. 700 µL of D/HY@PNE were added to dialysis bags (molecular weight cut off =3500 Da) and dialyzed in 25 mL PBS at pH 7.4, 6.5, or 5.0 in the presence of 0.5% (W/V) Tween-80 with or without GSH (2 µM or 10 mM) at 37 °C under shaking at 180 rpm. At defined time intervals, 1.0 mL of release media was removed and 1.0 mL of fresh release media was added. The DOX concentrations of different samples were determined by fluorophotometer (FL-4500, Hitachi, Japan) and HY concentrations were detected by HPLC (HP-1100, Agilent, USA).

### Cell uptake analysis

4T1 cells were seeded in Petri dishes at a density of 2×10^5^ cells/dish. 24 h later, the medium was then replaced by the culture medium with D@PNE (10 µg/mL) at pH 7.4 or 6.5 and incubated for 2 h. Subsequently, cells were washed twice with PBS and fixed in 4% paraformaldehyde stationary solution for 15 min. Thereafter, cells were washed twice with PBS, and stained with DAPI (2.5 µg/mL) for 8 min. After washing with PBS, images were taken by a CLSM (FV1000, Olympus, Japan). The cellular fluorescence intensity was also quantitatively analyzed by flow cytometry (Cytoflex, Beckman Coulter, USA). The distribution of DOX fluorescence was analyzed using Image J software.

### Cytotoxicity analysis

The cytotoxicity was detected by a CCK-8 kit. 4T1 cells and NIH-3T3 cells were cultured in 96-well plates at 5×10^3^ cells /well. NIH-3T3 cells were incubated in the culture medium with different PNE concentrations for 24 h. 4T1, A549, and B16 cells were incubated in the culture medium with D@PNE at pH 7.4 or 6.5 for 24 h. Untreated cells served as controls. The absorbance was measured at 450 nm using a microplate reader (318C- microplate reader).

### Detection of extracellular release of HMGB-1 and ATP

4T1 cells were cultured on 24-well plates at a density of 5×10^4^ cells /well. After 24 h, the medium was replaced with the culture medium containing PNE (25 µg/mL), free DOX (1 µg/mL), and D@PNE (1 µg/mL) at pH 7.4 or 6.5. Untreated cells served as the control group. After 2 h, the HMGB-1 and ATP contents in supernatants were detected by HMGB-1 ELISA and ATP bioluminescence assay kits, respectively.

### CRT exposure test *in vitro*

4T1 cells were cultured in 6-well plates at a density of 5×10^5^ cells/well for 24 h. The medium was then replaced by the culture medium with PNE (25 µg/mL), free DOX (1 µg/mL), D@PNE (1 µg/mL) at pH 7.4 or 6.5. Four hours later, cells were collected and incubated with the mouse CRT antibody (0.25 µg/10^6^ cells) for 40 min. After washing three times with cold PBS, cells were incubated with the Alexa 647-conjugated goat anti-mouse IgG antibody (2.5 µg/10^6^ cells) for 20 min and then tested by flow cytometry to identify cell surface CRT.

### Inducing DC maturation *in vitro*

To monitor DC maturation* in vitro*, BMDCs used as immature DCs (iDCs) were obtained from the bone marrow of 8-week-old BALB/c mice. 4T1 cells were pretreated with DOX or D@PNE for 24 h at a DOX concentration of 1 µg/mL. Subsequently, 5×10^5^ iDCs were co-cultured with pretreated 4T1 cells for 24 h. After incubation with anti-CD11c-PE/Cy7, anti-CD80-PE, and anti-CD86-APC antibodies, DC maturation was examined by using flow cytometry.

### Penetration of D/HY@PNE

4T1 MCSs and *ex vivo* tumors were used to examine the penetration depth of D/HY@PNE. The three-dimensional 4T1 MCSs were cultured according to the method described previously 47, 48. Briefly, the fibrinogen/cell mixtures were acquired by mixing 2 mg/mL fibrinogen with the same volume of 4T1 cell suspension (4×10^3^ cells/mL). Subsequently, 50 µL fibrinogen/cell mixtures were seeded into each well of 96-well plates preloaded with 1 µL thrombin (0.1 U/µL). After incubation for 15 min, 250 µL RPMI 1640 medium was added. The tumor spheroids based on 4T1 cells were allowed to grow for 5 days.

The 4T1 MCSs were transferred to Petri dishes and incubated in RPMI 1640 medium containing DOX or D/HY@PNE with the total DOX concentration of 5 µg/mL at pH 7.4 or 6.5. After incubation for 5 h, the spheroids were harvested, washed with PBS three times, and imaged using an Olympus FV1000 confocal microscope. The distribution of DOX fluorescence was analyzed using Image J software.

For the *ex vivo* tumor penetration, 4T1 tumor tissues were excised from 4T1 tumor-bearing mice and then incubated in RPMI 1640 medium containing DOX or D/HY@PNE with the total DOX concentration of 5 µg/mL at pH 7.4 or 6.5. After incubation for 24 h at 37 °C, the tumors were washed with PBS, followed by cryotomy. The DOX fluorescence of the frozen tumor sections was imaged using a Pannoramic Desk (P1000, 3DHIESTECH, Hungary). The distribution of DOX fluorescence from the tumor periphery to the center was analyzed using Image J software.

### *In vivo* antitumor effect of D/HY@PNE

4T1 cells (2×10^6^) were subcutaneously injected into the flanks of female Balb/c mice (6-8 weeks, 17-20 g). 4T1 tumor bearing Balb/c mice were randomly divided into 6 groups (n = 5). When the tumor volume reached 60 mm^3^, PBS, PNE, DOX, HY, DOX+HY and D/HY@PNE were intratumorally injected every 2 days 3 times with the dose of 0.9 mg/kg DOX and 0.83 mg/kg HY. The tumor volume and body weight were recorded every day. The tumor volume was calculated as follows: V=L×W^2^/2 (L, the longest dimension; W, the shortest dimension). After 12-day treatment, mice were sacrificed. Then, sera, tumor tissues and the main organs were collected. The tumor tissues and the main organs were fixed with 4% paraformaldehyde and sliced for H&E and Ki67 staining following standardized protocols 16. The fixed tumor sections were also subjected to immunofluorescence assay of CRT and HMGB-1 according to the method described previously 16, 49 and imaged using a Pannoramic Desk (P1000, 3DHIESTECH, Hungary).

### *In vivo* immune response analysis of the combined therapeutics

To examine immune responses after various treatments, CD8^+^ T cell population, MDSC (CD11b^+^Gr-1^+^), and Treg (Foxp3^+^) levels in tumor regions were analyzed using immunofluorescence staining. CD4^+^ and CD8^+^ T cells in tumors were also analyzed using flow cytometry. Tumors were cut into small pieces and immersed in 0.8 mg/mL type I collagen solution for 30 min at 37 °C, and the small tumor pieces were pressed gently to obtain a single-cell suspension. The single cells were further stained with several fluorochrome-conjugated antibodies: anti-CD45-APC/Cy7, anti-CD3-FITC, anti-CD4-BV421, anti-CD8-PE/Cy7, and anti-PD-L1-APC, and then analyzed by flow cytometry. Besides, the cytokines IFN-γ and TNF-α in sera were tested by ELISA following the manufacturer's instructions.

To detect the DC maturation, tumor-bearing mice were treated as described above. The tumor-draining lymph nodes were harvested on day 3 after the first treatment and pressed gently to obtain a single-cell suspension. Cells were stained with anti-CD11c-PE/Cy7, anti-CD80-PE, and anti-CD86-APC using standard protocols and further analyzed by flow cytometry.

### Statistical analysis

Data were presented as mean ± SEM. Significance was determined by the Student's t-test for the comparison of two groups and one-way ANOVA for multiple groups. The differences were considered significant for *p < 0.05 and very significant for **p < 0.01 or ***p < 0.001.

## Supplementary Material

Supplementary figures.

## Abbreviations

ICP: immune checkpoint
DOX: doxorubicin
ICD: immunogenic cell death
PD-1: programmed cell death protein 1
PD-L1: programmed cell death protein-ligand 1
HY: HY19991
IFN-γ: interferon-γ
GSH: glutathione
IPM: isopropyl myristate
DCs: dendritic cells
iDC: immature DC
DLS: dynamic light scattering
PNE: Pickering nanoemulsion
MFI: mean fluorescence intensity
LCST: lower critical solution temperature
TEM: transmission electron microscope
PDI: polydispersity index
CLSM: confocal laser scanning microscopy
CCK-8: cell counting Kit-8
IC_50_: half-maximal inhibitory concentration
DLC: drug loading capacity
CRT: calreticulin
HMGB-1: high mobility group box 1
MCSs: multicellular spheroids
ATP: adenosine triphosphate
MDSCs: myeloid-derived suppressor cells
Tregs: regulatory T cells
BMDCs: bone marrow-derived dendritic cells
TNF-α: tumor necrosis factor-α
