# Supplementary material for: A pH-responsive Pickering Nanoemulsion for specified spatial delivery of Immune Checkpoint Inhibitor and Chemotherapy agent to Tumors
Source: Theranostics. 2020 Aug 7;10(22):9956–69. doi: 10.7150/thno.46089 (PMC7481420; doi:10.7150/thno.46089)
Supplement: Supplementary file 1 — Supplementary figures. [file thnov10p9956s1.pdf]

Electrical Supporting information for

A pH-responsive Pickering nanoemulsion for specified spatial delivery of immune checkpoint inhibitor and chemotherapy agent to tumors

Le Jia[1], Minghui Pang[2], Man Fan[2], Xuan Tan[1], Yiqian Wang[1], Menglin Huang[1], Yijing Liu[2], Qin Wang[2]*, Yanhong Zhu[1]*, Xiangliang Yang[1]

[1]National Engineering Research Center for Nanomedicine, College of Life Science and Technology, Huazhong University of Science and Technology, Wuhan 430074, China

[2]Hubei Key Laboratory of Bioinorganic Chemistry and Materia Medica, School of Chemistry and Chemical Engineering, Huazhong University of Science and Technology, Wuhan 430074, China

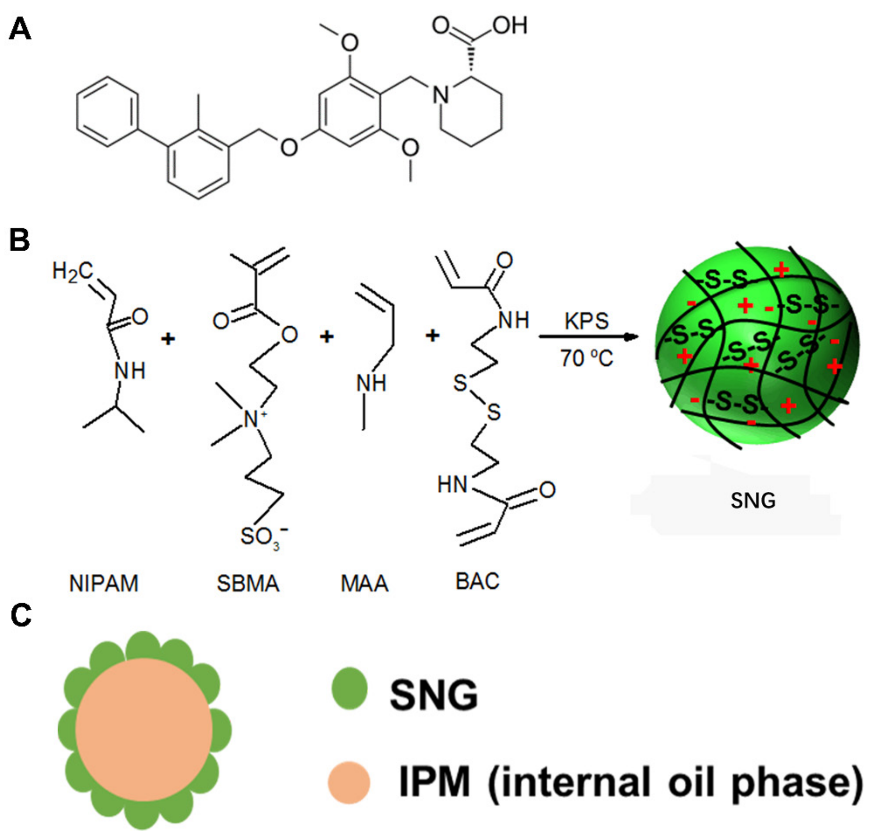

Figure S1. (A) Chemical structure of HY. (B) Synthesis of SNG. (C) Schematic representation of blank PNE.

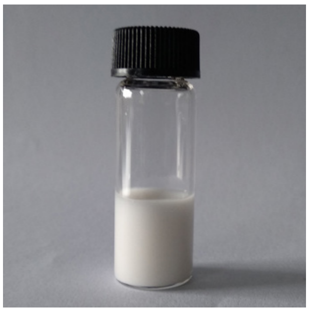

Figure S2. Photo of the blank PNE.

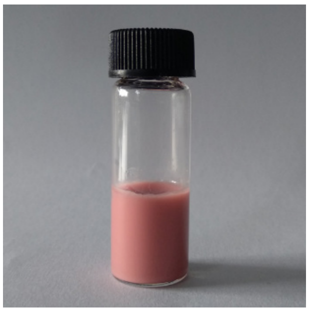

Figure S3. Photo of D/HY@ PNE after fabrication immediately.

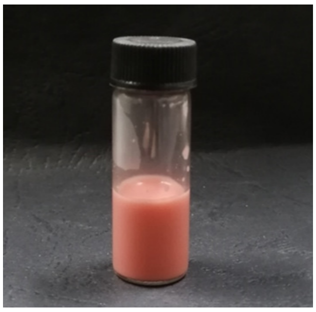

Figure S4. Photo of D/HY@ PNE stored at 4 °C for 6 months.

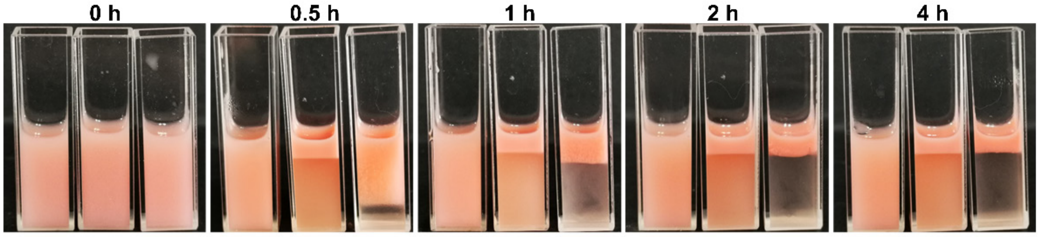

Figure S5. Photos of D/HY@PNE with different pH warmed at 37 °C for different time. From left to right, the pH value is 7.4, 6.5 and 5.0, respectively.

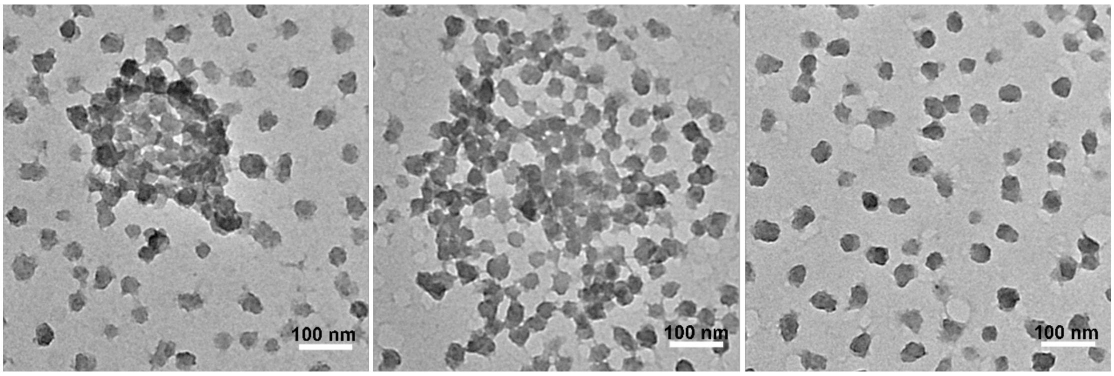

Figure S6. TEM images of PNE with pH 6.5 warmed at 37 °C for 4 h taken at different visual field.

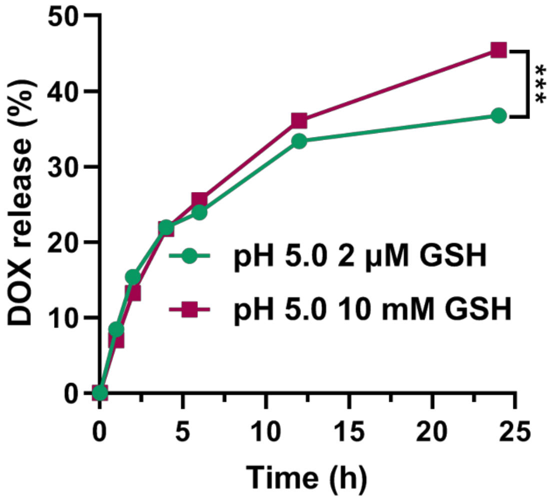

Figure S7. Cumulative released DOX from D/HY@PNE in different concentration of GSH at different time points.

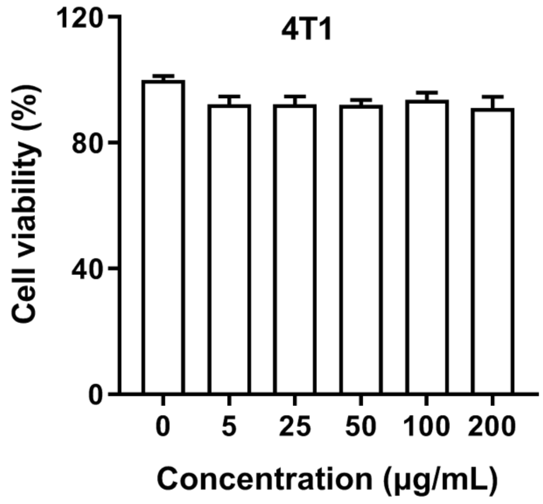

Figure S8. The cell viability of 4T1 cells incubated with PNE for 24 h.

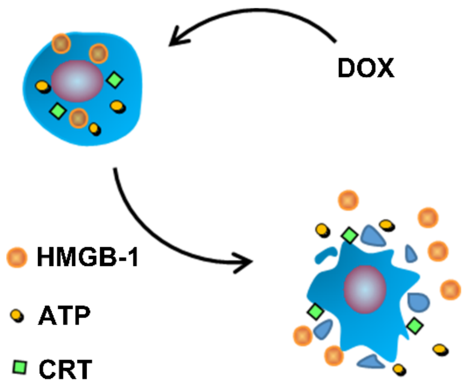

Figure S9. Schematic illustration of DOX-induced ICD of tumor cells as characterized by CRT exposure, ATP secretion, and HMGB-1 release.

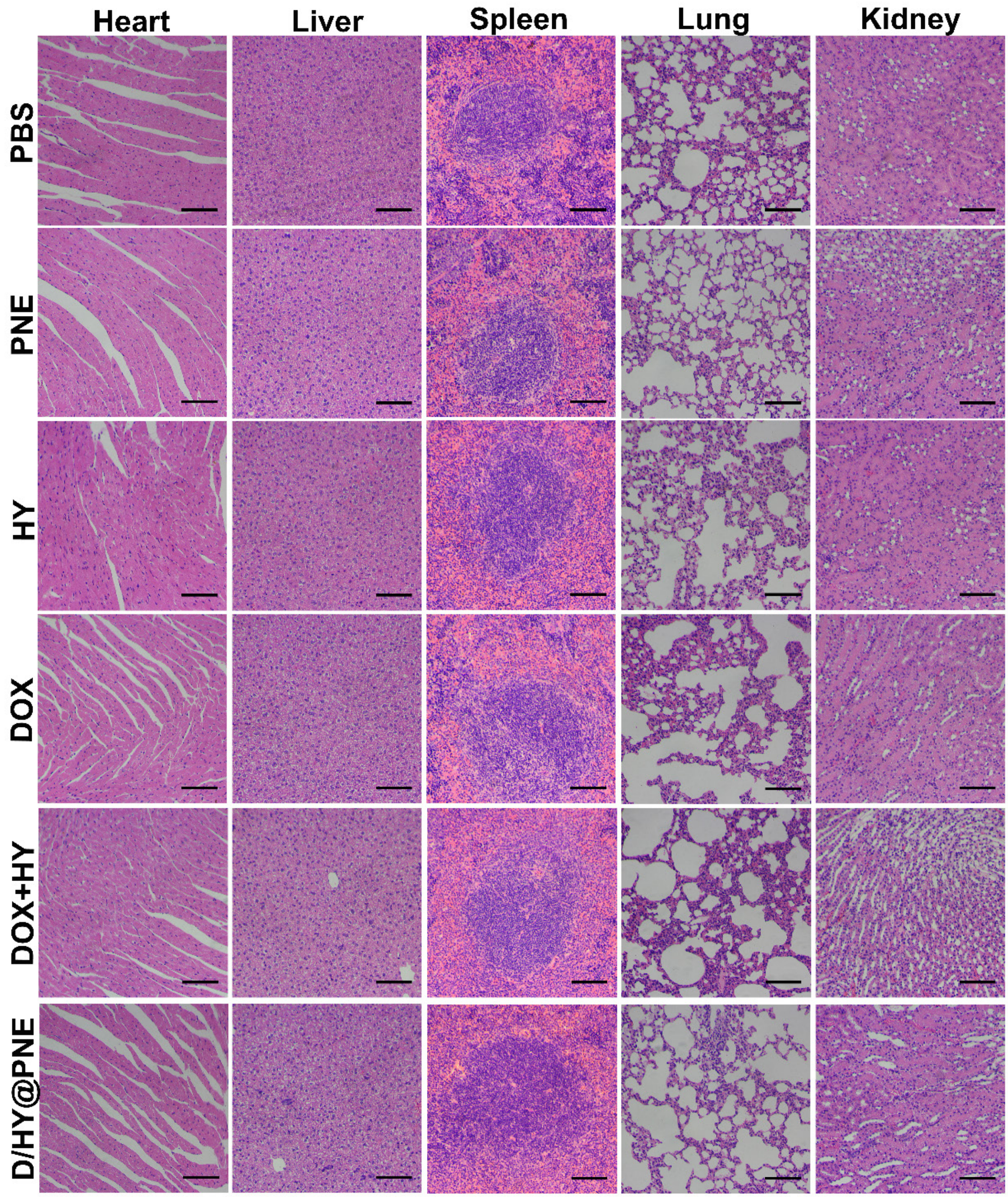

Figure S10. H&E staining of the major organs of 4T1-bearing mice at the end of antitumor studies. The scale bar is 50 μm.

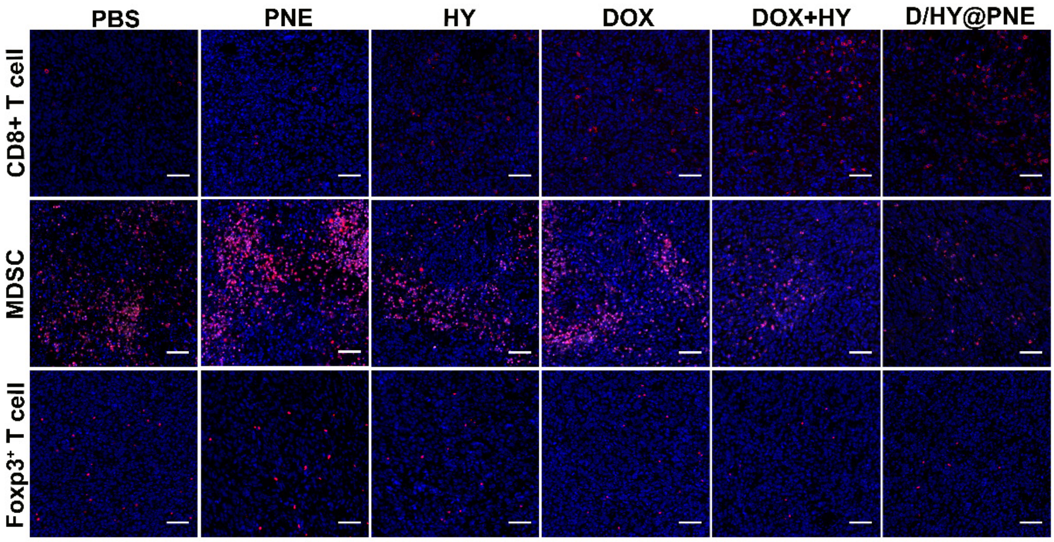

Figure S11. Immunofluorescence staining was used to test CD8 $^{+}$ T cells, MDSCs ($CD11b^{+}Gr\text{-}1^{+}$) and Tregs ($Foxp3^{+}$) infiltration in tumor sections at the end of the treatments. The scale bar is 50 μm.
